# Interactive network-based clustering and investigation of multimorbidity association matrices with associationSubgraphs

**DOI:** 10.1093/bioinformatics/btac780

**Published:** 2022-12-06

**Authors:** Nick Strayer, Siwei Zhang, Lydia Yao, Tess Vessels, Cosmin A Bejan, Ryan S Hsi, Jana K Shirey-Rice, Justin M Balko, Douglas B Johnson, Elizabeth J Phillips, Alex Bick, Todd L Edwards, Digna R Velez Edwards, Jill M Pulley, Quinn S Wells, Michael R Savona, Nancy J Cox, Dan M Roden, Douglas M Ruderfer, Yaomin Xu

**Affiliations:** Department of Biostatistics, Vanderbilt University, Nashville, TN 37232, USA; Department of Biostatistics, Vanderbilt University, Nashville, TN 37232, USA; Department of Biostatistics, Vanderbilt University, Nashville, TN 37232, USA; Vanderbilt Genetics Institute, Vanderbilt University Medical Center, Nashville, TN 37232, USA; Division of Genetic Medicine, Department of Medicine, Vanderbilt University Medical Center, Nashville, TN 37232, USA; Department of Biomedical informatics, Vanderbilt University Medical Center, Nashville, TN 37232, USA; Department of Urology, Vanderbilt University Medical Center, Nashville, TN 37232, USA; Vanderbilt Institute for Clinical and Translational Research, Vanderbilt University Medical Center, Nashville, TN 37232, USA; Division of Hematology and Oncology, Department of Medicine, Vanderbilt University Medical Center, Nashville, TN 37232, USA; Division of Hematology and Oncology, Department of Medicine, Vanderbilt University Medical Center, Nashville, TN 37232, USA; Department of Medicine, Center for Drug Safety and Immunology, Vanderbilt University Medical Center, Nashville, TN 37232, USA; Institute for Immunology and Infectious Diseases, Murdoch University, Murdoch, WA 6150, Australia; Division of Genetic Medicine, Department of Medicine, Vanderbilt University Medical Center, Nashville, TN 37232, USA; Division of Epidemiology, Department of Medicine, Vanderbilt University Medical Center, Nashville, TN 37232, USA; Department of Obstetrics and Gynecology, Vanderbilt University Medical Center, Nashville, TN 37232, USA; Vanderbilt Institute for Clinical and Translational Research, Vanderbilt University Medical Center, Nashville, TN 37232, USA; Department of Allergy, Pulmonary and Critical Care Medicine, Vanderbilt University School of Medicine, Nashville, TN 37232, USA; Vanderbilt Genetics Institute, Vanderbilt University Medical Center, Nashville, TN 37232, USA; Department of Cardiovascular Medicine, Vanderbilt University Medical Center, Nashville, TN 37232, USA; Department of Internal Medicine, Vanderbilt University Medical Center, Nashville, TN 37232, USA; Vanderbilt Genetics Institute, Vanderbilt University Medical Center, Nashville, TN 37232, USA; Division of Genetic Medicine, Department of Medicine, Vanderbilt University Medical Center, Nashville, TN 37232, USA; Department of Pharmacology, Vanderbilt University Medical Center, Nashville, TN 37232, USA; Vanderbilt Genetics Institute, Vanderbilt University Medical Center, Nashville, TN 37232, USA; Division of Genetic Medicine, Department of Medicine, Vanderbilt University Medical Center, Nashville, TN 37232, USA; Department of Biomedical informatics, Vanderbilt University Medical Center, Nashville, TN 37232, USA; Department of Psychiatry and Behavioral Sciences, Vanderbilt University Medical Center, Nashville, TN 37232, USA; Department of Biostatistics, Vanderbilt University, Nashville, TN 37232, USA; Department of Biomedical informatics, Vanderbilt University Medical Center, Nashville, TN 37232, USA

## Abstract

**Motivation:**

Making sense of networked multivariate association patterns is vitally important to many areas of high-dimensional analysis. Unfortunately, as the data-space dimensions grow, the number of association pairs increases in *O*(*n*^2^); this means that traditional visualizations such as heatmaps quickly become too complicated to parse effectively.

**Results:**

Here, we present associationSubgraphs: a new interactive visualization method to quickly and intuitively explore high-dimensional association datasets using network percolation and clustering. The goal is to provide an efficient investigation of association subgraphs, each containing a subset of variables with stronger and more frequent associations among themselves than the remaining variables outside the subset, by showing the entire clustering dynamics and providing subgraphs under all possible cutoff values at once. Particularly, we apply associationSubgraphs to a phenome-wide multimorbidity association matrix generated from an electronic health record and provide an online, interactive demonstration for exploring multimorbidity subgraphs.

**Availability and implementation:**

An R package implementing both the algorithm and visualization components of associationSubgraphs is available at https://github.com/tbilab/associationsubgraphs. Online documentation is available at https://prod.tbilab.org/associationsubgraphs_info/. A demo using a multimorbidity association matrix is available at https://prod.tbilab.org/associationsubgraphs-example/.

## 1 Introduction

Analysis of association or correlation between variables is a very important step in exploratory data analysis of high-dimensional datasets. In these scenarios, a dataset with a large number of columns or variables, but without known or validated patterns of association among them, can be represented as an undirected weighted network with edges having a weight proportional to the strength of pairwise variable associations (Hevey, 2018). To construct such a network, it is necessary to incorporate a statistical assessment of the associations. The issues of sampling, measurement error or confounding effect are of potential concern, and it is often hard to provide an optimal criterion to differentiate true versus false associations. It is highly desirable that this network can be inspected using statistical and visualization methods to gain insight into how the variables correlate with each other. There are many different ways of establishing the strength of these associations, from as simple as the mutual occurrence of binary variables (Cha, 2007), to complex penalized regression models (Hallac *et al.*, 2015; Tibshirani *et al.*, 2005). Examples of areas where association analysis is used include the following: gene regulatory networks (Gustafsson *et al.*, 2005), analysis of single-cell sequencing data to determine cell differentiation (Chan, 2017), networks of comorbidity between diseases (Chen and Xu, 2014) and topic modeling in natural language processing (Wang and Zhu, 2014).

The traditional analysis visualizing these networked association patterns uses heatmaps. In these visualizations, both rows and columns represent all present variables, and the color of the cells represents the strength of the association between the two variables. As the number of variables grows larger, the effectiveness of heatmaps rapidly decreases. One important issue is the ordering of the rows and columns, as the precise placement of a variable in relation to other completely changes inference made by the analyst (Bojko, 2009) and thus must be done carefully. Typically, this ordering is done by a clustering algorithm (Metsalu and Vilo, 2015; Pryke *et al.*, 2007) that injects model assumptions into the visualization, which are not immediately clear to the analyst or later audience. Another issue with large heatmaps is the difficulty of discerning the identity of cells that fall far from the labeled axes (Bojko, 2009).

Another way to investigate the networked association patterns is to reduce the association space using edge filtering and visualize the results using network clustering. One such method is the so-called single-linkage clustering, in which a network—whose nodes are the set of variables, where two such variables are connected by an edge if their weight (association strength) is greater than a given threshold (Altman and Krzywinski, 2017)—is constructed. The goal is to investigate sets of ‘similar’ subgraphs when an optimal cutoff value is provided. These methods require making many decisions manually, such as a reasonable cutoff value, the number of groups and the statistical model to be used. We are commonly left with multiple candidates with seemingly reasonable results and the user has to decide which inferred clustering should be regarded as informative. In addition, these methods typically involve parametric tests for node associations that do not account for the network structure (Benjamini and Yekutieli, 2001; Simas *et al.*, 2021) or contain many assumptions (Hallac *et al.*, 2015). Non-parametric methods typically based on permutations also exist but, due to the *O*(*n*^2^) complexity inherent in association datasets, are computationally infeasible for large datasets (Harris and Drton, 2013). Some work in clustering theory has been done in trying to determine the optimal cutoff values (Flake *et al.*, 2004), but it is now understood that it is much more informative to maintain the entire clustering dynamics (e.g. the entire dendrogram) and provide a summary of the behavior of clustering under all possible cutoff values at once. We point the reader to the book by Kolaczyk and Csárdi (2020) for more information on these methods.

Network percolation is a process that some fraction of the nodes, along with the edges connected to those nodes, are removed from a network. It is used to investigate the structural resilience of the networks and as a benchmark model for understanding the network dynamics such as epidemic spreading, vital node identification and network clusters (Arias-Castro and Grimmett, 2013; Derényi *et al.*, 2005; Lü *et al.*, 2016; Newman, 2002). Percolation theory (Newman, 2018) is a subfield of network science dedicated to understanding the percolation process and how the removal of nodes and their edges within a network leads to the formation of ‘isolated subgraphs’ (also called ‘components’ or ‘clusters’). Here, an isolated subgraph refers to a group of nodes connected internally but not to any other nodes in the network (henceforth referred to as the subgraph). In this article, we visualize the percolation of an association network and provide a dynamic view of the subgraph structure by removing/adding edges and nodes according to a strength measurement that is obtained from the statistical assessment of pairwise associations between variables. In this view, users can quickly examine informative subgraphs at different cutoff values of the association strength, examine the evolvement of the subgraphs as a function of association strength when, for example, the edges and nodes are removed in order of strength from lowest to highest, evaluate the robustness of the resulting subgraphs as we adjust the cutoff values and identify potential phase transition of the network with important subgraphs. The ‘random graph’ (Solomonoff and Rapoport, 1951) (also called the Erdos–Renyi graph), a classic null model studied in network sciences, is often used in network percolation in which nodes were removed uniformly at random without preference for any specific nodes or according to any specific order. This configuration is mainly used as a naive reference model to understand the behavior of percolation in real-world networks when nodes are removed according to any user-specified node-removal scheme.

Disease multimorbidity represents the association of multiple disorders due to co-existing conditions in patients. Understanding the disease multimorbidity may shed light on shared molecular mechanism and/or environmental exposure of the comorbid diseases or their interplay. We previously developed PheWAS-ME: an interactive dashboard to visualize individual-level genotype and phenotype data side-by-side with PheWAS analysis results, allowing researchers to explore multimorbidity patterns and their associations with a genetic variant of interest (Strayer *et al.*, 2021). In this work, by framing association analysis of disease multimorbidity as a network problem, we can utilize the results of percolation theory to design an intuitive set of visualizations for exploring the subgraph structure of multimorbidity based on the concept of adding and removing edges in the order of the strength of association. We name this algorithm associationSubgraphs and implement it as an interactive visualization method to quickly explore high-dimensional association datasets. As a use case example, we applied associationSubgraphs to a phenome-wide multimorbidity association matrix generated from an electronic health record (EHR) and provide an online, interactive demonstration for exploring multimorbidity subgraphs.

**Algorithm 1**. Find neighborhood subgraphs. By simply requiring the association strengths to be sorted, the only assumption required of the strength measure is monotonicity.

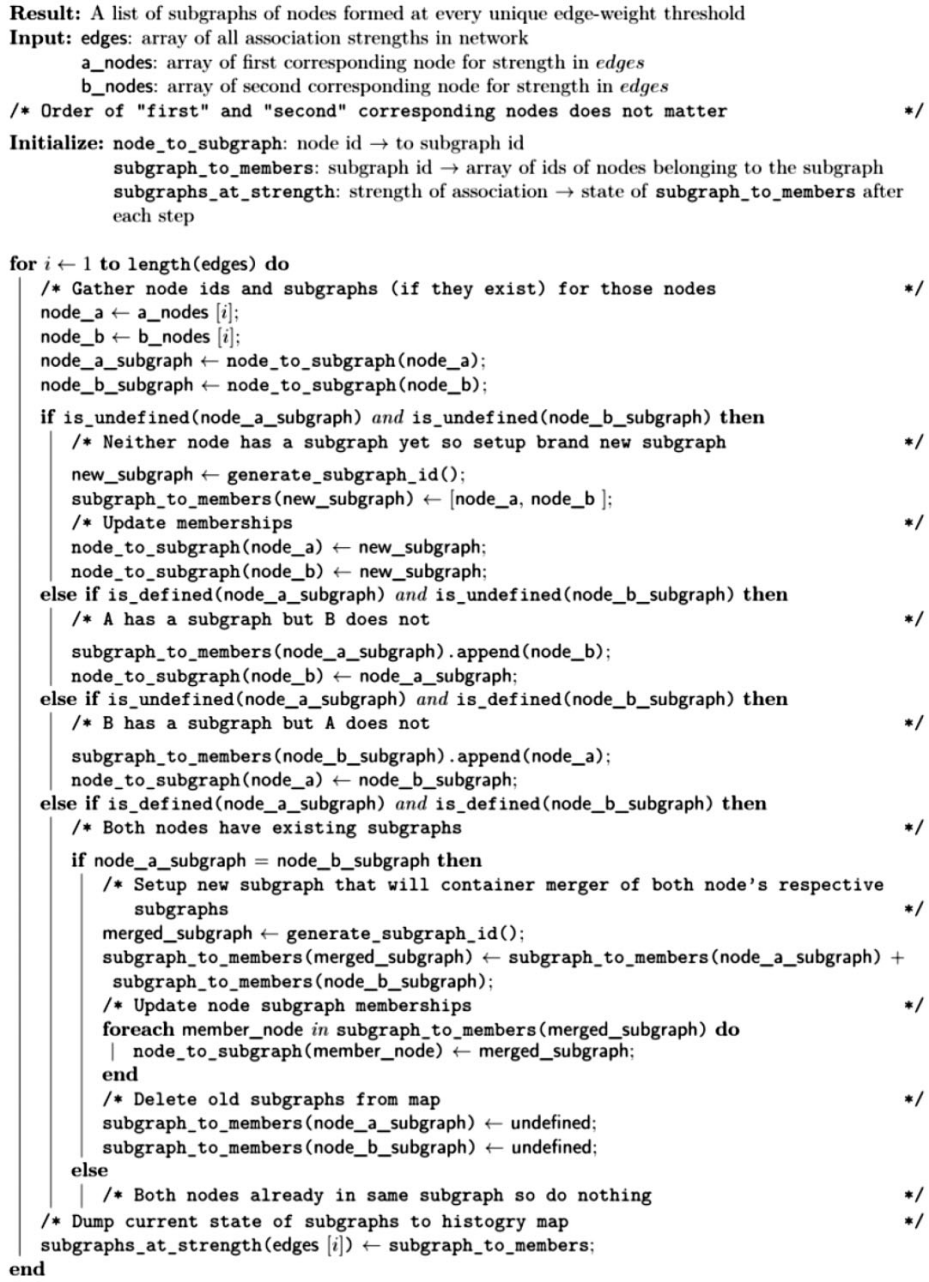

## 2 Materials and methods

### 2.1 Algorithm

The algorithm for computing associationSubgraphs at all given cutoffs is closely related to single-linkage clustering (Gower and Ross, 1969) but differs philosophically by viewing nodes that are yet to be merged with other nodes as unclustered rather than residing within their own cluster of size one.

The algorithm calculates the set of subgraphs at every threshold by connecting the two nodes with the highest association strength as a ‘cluster’ of size two. The next highest association, ‘edge’, is then considered. If one of the edge’s nodes is already in a cluster, then the other node is added to that cluster. If both nodes exist in separate clusters, those two clusters are merged into a new ‘super’ cluster. If both nodes are not in any cluster, they form a new size-two cluster. Lastly, no changes are made if both nodes already exist in the same cluster. This process continues for every association edge, with each step providing the clustering state up to the threshold just below the most recently added edge’s strength. For further details, see Algorithm 1.

### 2.2 Visualization

The subgraph-clustering algorithm results are visualized through an interactive visualization built using the javascript library D3 (Bostock *et al.*, 2011) that allows panning through and visualizing all cluster states that occur during the running of the algorithm.

**Fig. 1.**
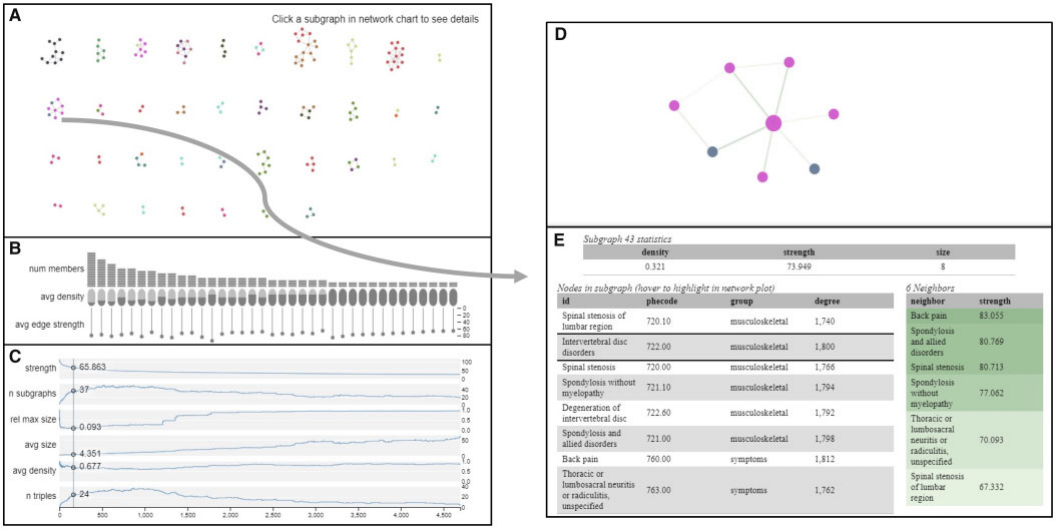
Interactive visualization of subgraph-clustering results with the current threshold set at the optimal threshold according to the smallest–largest rule. (**A**) Clustered nodes displayed as a grid of force-directed subgraphs. (**B**) Chart with three network measures: number of members (nodes), average density and average edge strength. (**C**) Line plots of edge association strength, number of subgraphs, relative maximum size, average size, average density, and number of triples. Slide to choose a different strength threshold to update the dynamic clustering in A. (**D**) An example subgraph after users click on it in A. (**E**) Meta-data and network neighbour information corresponding the selected subgraph in D

At every step, all currently clustered nodes are displayed as a grid of force-directed subgraphs (de Leeuw, 1988) (Fig. 1A). Accompanying each subgraph is a set of three measures as encoded in a chart (Fig. 1B). These are the number of nodes in the subgraph, the density (number of edges at current threshold linking nodes relative to the maximum possible number of edges without the threshold), and the average strength of all those edges.

By adding edges in one-at-a-time, we are emulating the formation of a random graph. As the edges are added, isolated subgraphs form within the network. By separating the subgraphs, the visualization acknowledges that, at the current association strength threshold, the nodes are conceptually isolated and should be represented as such, unlike traditional network or heatmap visualizations.

To aid in the selection of the association threshold, a series of line plots below the network visualization (Fig. 1C) provide summary statistics about the cluster state at every possible cutoff. These include the number of subgraphs, the number of subgraphs with at least three members (triples), the average density of those subgraphs, the average size of the subgraphs, the size of the largest subgraph relative to all other subgraphs combined and the current association threshold. By hovering over a state in these line plots, the drawn network updates to represent the desired threshold. This updating is done in real-time allowing the user to see exactly what edges were added and how those edges changed the subgraph state.

Every resulting subgraph can be selected and zoomed into by clicking, which reveals all members within the cluster (Fig. 1D), the edge strengths between them, and any further supplementary node information provided by the user (Fig. 1E).

### 2.3 Choosing ‘optimal’ threshold

AssociationSubgraphs is meant to provide an exploratory view of an entire association network; this means the concept of the ideal threshold is not particularly important. However, we can draw inspiration from random graph and percolation theory to provide an estimate of an ‘optimal’ threshold value used as the initial point in the visualization.

When nodes are truly randomly connected together, what is known as a ‘giant component’ forms very quickly. A giant component is one in which a very large portion, typically *n*^2/3^ (Mark Newman, 2018), of the nodes in the network are in the same isolated subgraph. There are three ‘phase-transitions’ regarding the size of the largest isolated subgraph in the network relative to the number of edges (*e*) added.

If e<n, we would expect many small subgraphs with the largest size on the order of log⁡(n). If e=n, then we would expect to still have a large number of subgraphs, with an expected largest subgraph of size *n*^2/3^. Last, if e>n we would expect all nodes to be connected in one giant subgraph/component.

When the edges are not purely random, we expect a deviation from these patterns, and in practice we see these deviations; with a giant component typically forming well after the number of included edges surpasses the number of nodes.

To take advantage of this known behavior, we propose the ‘largest–smallest’ rule for finding the optimal threshold. This rule states that the optimal threshold value for an association network will be just before the giant component starts to form. This point is estimated by tracking the aforementioned size of the largest subgraph relative to the combined size of all other subgraphs. When this metric starts to rise, it indicates that edges are now being added mostly at random. Thus, the optimal threshold can be seen as the minimum of this function.

### 2.4 Multimorbidity network

Multimorbidity, defined as the co-existence of two or more concurrent health conditions in one patient, can be represented as networks with diseases as nodes and their connections as links that are typically weighted according to the strength of pairwise disease associations. Figure 1 shows the results of running the associationSubgraphs algorithm and visualization on such a multimorbidity network of 1815 phenotypes as ‘Phecodes’ (Denny *et al.*, 2010) constructed using Vanderbilt’s EHR data. The network strength measurements are based on the test statistics of a regression analysis assessing the association between each Phecode pairs. AssociationSubgraphs provides intuitive and meaningful insights into the subgraph structure of the example multimorbidity network. Using the described smallest–largest point to determine an association strength, cutoff returns a network with 36 isolated subgraphs. Figure 1E shows the investigation of one of the present subgraphs, including the codes 720.00, 720.10, 721.00, 721.10, 722.00, 722.60, 760.00 and 763.00, which are all back pain-related Phecodes (e.g. Spinal stenosis of the lumbar region: 720.10 and Back pain: 760.00) More examples and this particular visualization are available on the associationSubgraphs R package website (see Availability and implementation).

### 2.5 Case study: multimorbidity analysis of kidney stone disease

**Fig. 2.**
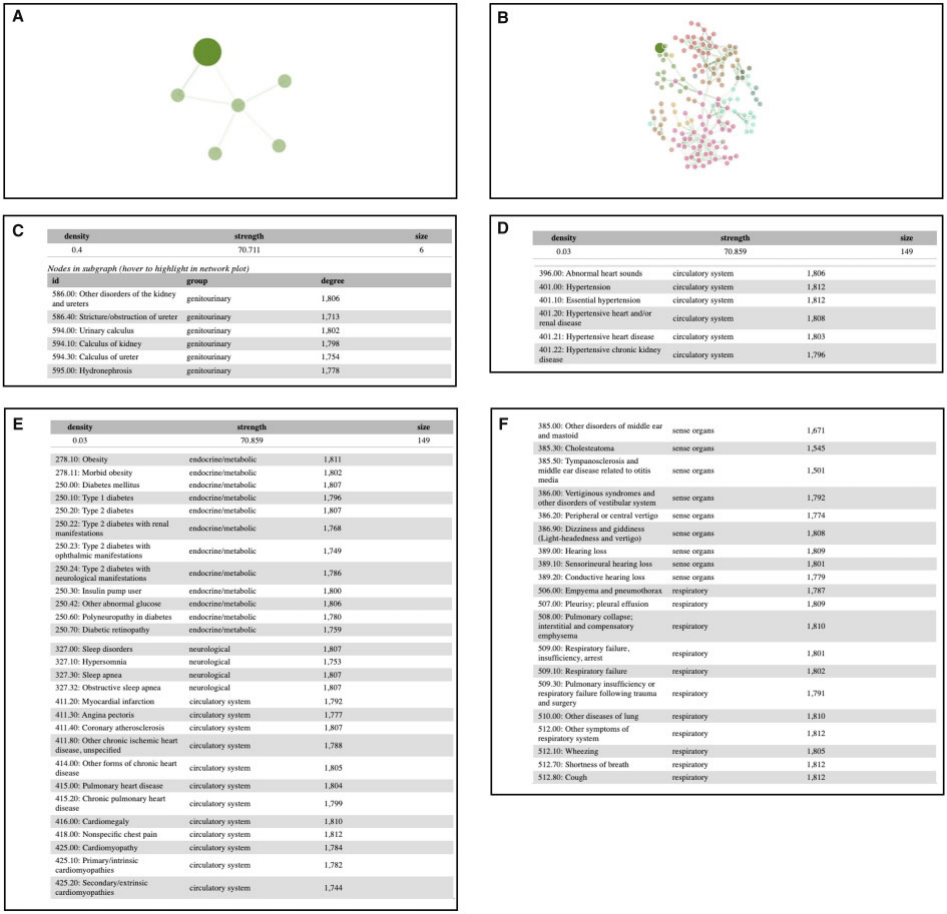
Multimorbidity analysis of kidney stone disease at different threshold values. (**A**) A focused view using a strength cutoff value 64.029. (**B**) A focused view using a strength cutoff value 55.54. (**C**) In the subgraph showing in A, Kidney stone disease is linked to neighboring diagnoses of urinary stones located in the kidney and ureter, and additionally with hydronephrosis and stricture. (**D**) In the subgraph in B, kidney stone disease is associated with hypertension related diagnoses. (**E**) Further link to obesity, diabetes mellitus, sleep apnea, and heart disease diagnoses. (**F**) Some of novel disease associations that are not currently considered linked to kidney stone pathogenesis, may represent potential areas for future discovery

Kidney stone disease (Phecode 594.10) is well characterized in the multimorbidity network framework both due to its clinical presentation and its disease associations. The condition is marked by acute, symptomatic episodes leading to clinical care over time. Under the multimorbidity network, we illustrate several use cases to discover the gradual disease multimorbidity patterns, and how some may lead to novel findings for downstream investigation. First, under a focused view using a strength cutoff value of 64.029 (example density 0.4, strength 70.711, size 6) (Fig. 2A and C), kidney stone disease is linked to neighboring diagnoses of urinary stones located in the kidney and ureter, and additionally is closely associated with hydronephrosis and stricture. These diagnoses co-occur commonly at or around the time of clinical presentation. For a given clinical encounter, an initial diagnosis may evolve into more refined diagnoses as additional clinical information is gained, for example, the diagnosis of renal obstruction, and then later the determination of a stone in the ureter causing the renal obstruction. Second, as we relax the strength criteria to 55.54 (Fig. 2B), we observe more associations in the surrounding nodes, and most of those are well known in the literature. We first observe hypertension-related diagnoses (Fig. 2D). These then link to obesity, diabetes mellitus, sleep apnea and heart disease diagnoses (Fig. 2D). Kidney stone disease has been linked to multiple systemic conditions, including chronic kidney disease (Rule *et al.*, 2009), hypertension (Madore, 1998), obesity (Taylor, 2005) and diabetes mellitus (Taylor *et al.*, 2005). Longitudinal studies have shown the association of kidney stone disease with the development of coronary heart disease (Ferraro *et al.*, 2013; Rule *et al.*, 2010). Taken together, the visualization patterns enable validation of known disease associations. Finally, nodes that are connected more peripherally may represent potential areas of future discovery. Conditions related to sleep, hearing and the vestibular system, and pulmonary disorders appear potentially related to kidney stone disease (Fig. 2E and F). To date, these organ systems are typically not considered to be linked to kidney stone pathogenesis, but these data from associationSubgraphs raise new questions and hypotheses on disease effectors or modifiers.

## 3 Conclusion

In this article, we have provided a brief introduction to the algorithm and visualization components of associationSubgraphs for exploring association networks. By enabling the exploration of high-dimensional association networks through interactive network visualization guided by the basic network-science theory, associationSubgraphs allows researchers to understand their association network data with greater precision and intuition. We applied associationSubgraphs to a large-scale disease multimorbidity association matrix generated from EHR and provided an online, interactive demo for exploring multimorbidity subgraphs.

## Data Availability

The data underlying the article are available online. See Abstract Availability for details.
